# A scalable distributed pipeline for reference-free variants calling

**DOI:** 10.1186/s12864-025-11722-7

**Published:** 2025-06-03

**Authors:** Lorenzo Di Rocco, Umberto Ferraro Petrillo

**Affiliations:** Department of Statistical Sciences, Sapienza University of Rome, Rome, Italy

**Keywords:** Computational genomics, Variants calling, Distributed computing

## Abstract

**Background:**

Precision medicine pipelines typically begin with variant calling to identify disease-related mutations for optimal treatment selection. Reference-free approaches assess variations in the genetic profiles of distinct individuals through the utilization of a *De Bruijn* graph. However, the timely analysis of large-scale sequencing data may be beyond the capabilities of single workstations, requiring alternative computational approaches.

**Results:**

We introduce the first-known distributed pipeline for detecting isolated SNPs (Single Nucleotide Polymorphisms), by leveraging the computational resources of multiple machines in parallel. Our pipeline efficiently analyzes large datasets thanks to the usage of a distributed *De Bruijn* graph representation. Furthermore, we introduce a cluster-driven algorithm to partition the *De Bruijn* graph across multiple independent machines according to the inner structure of the sequences under analysis, thus further improving the scalability of our pipeline.

**Conclusions:**

The results of our experiments, conducted on real-world datasets, show the good performance of our pipeline in terms of efficiency, output quality and scalability. Moreover, the reported results also confirm that the adoption of a specialized partitioning algorithm for the distributed representation of the *De Bruijn* graph leads to a relevant performance speed-up compared to using standard partitioning techniques.

## Background

Advances in Next-Generation Sequencing (NGS) technologies have led to an explosion in the amount of omics data at affordable costs [1]. Due to this massive production of biological datasets, pivotal tasks in Bioinformatics have become computationally expensive.

One of the main computational challenge arises when performing large-scale analysis for *variants calling*. By this term we refer to a procedure aimed at detecting variants between the genomes of two different individuals. *Reference-based* algorithms are often used to identify the presence of mutations in the genetic makeup of an individual with respect to the average genome of the corresponding species [2].

But, high-quality reference genomes are not available for all species and for all applications. For these reasons, more recent solutions consider *reference-free* pipelines. These allow a comparison among different individuals leveraging exclusively on the corresponding raw samples of reads. However, the computational and memory requirements of these algorithms can be prohibitive, making them impractical for large dataset analysis on a single workstation.

Our goal is to take advantage of distributed computing to run reference-free variants calling pipelines at a large scale and in a feasible amount of time. More specifically, we propose a distributed pipeline for detecting isolated SNPs, i.e., those polymorphisms *k*-bases apart from other variants, where *k* is a fixed constant. Our approach, formulated according to the *MapReduce* paradigm and implemented with the Apache Spark framework, uses a distributed *De Bruijn* graph for fast and scalable *SNPs* calling from a very large collection of reads.

The effectiveness of our pipeline has been confirmed by several experiments we have conducted to evaluate its performance in terms of accuracy and scalability. Yet, the results of our experiments demonstrated also the existence of a performance bottleneck in our pipeline due to the standard strategy used by Spark for partitioning over a distributed system the vertices of an input graph. To address this problem, we introduce a partitioning algorithm based on hierarchical clustering and the Jaccard index for similarity measurement, able to optimize the distribution of graph vertices for the particular case of *SNPs* calling.

### Variants calling

Variants calling is a pivotal initial step in many bioinformatics analyses. It aims to identify mutations in the genome of an organism, including single nucleotide polymorphisms (*SNPs*), deletions or insertions of short nucleotide fragments (*indels*), and large structural variations (*SVs*).

Assessing the presence of a variant is challenging because modern technologies cannot reconstruct the entire genome of an individual in a pass, instead returning a huge chaotic set of redundant nucleotide fragments (called *reads*^1^). In addition, the reads are randomly sampled from one of the two strands and are prone to sequencing errors. Therefore, variants detection and genotyping usually require the alignment of the reads along a reference genome $G_{ref}$, made of characters drawn from the alphabet $\Sigma =\{A,C,G,T\}$, which implies a significant computational cost (i.e., an *alignment-based* approach).

More recent techniques avoid the alignment step and allow inference of the genotype by processing the raw reads directly (i.e., an *alignment-free* (AF) approach). We distinguish two main approaches.

#### Reference-based AF algorithms

These algorithms typically work by extracting all the substrings of length *k*, here called k-mers, from $G_{ref}$. The same is done for $G_{alt}$, i.e., the set of alternative sequences obtained by substituting in $G_{ref}$ all possible combinations of variants stored in an external file.

The k-mers extracted from $G_{ref}$ are referred to as *reference* k-mers, while those extracted from $G_{alt}$ are referred to as *alternative* k-mers. In both cases, the k-mers are stored using their *canonical representation* to allow strand-neutral comparison. The canonical k-mers are obtained by taking the lexicographically smaller string between a k-mer and its reverse complement occurring in the opposite nucleotide strand. All of this is done under the assumption that a k-mer represents a specific DNA fragment when *k* is sufficiently large to minimize the generation of substrings that occur in multiple regions of the genome (i.e., *ambiguous* k-mers). In this way, k-mers support the mapping of a read to a particular region or the evaluation of the coverage level for a polymorphism within the input sample.

Then, a statistical framework is used to examine the occurrences of $b_{ref}$ and $b_{alt}$, assigning each variant to a label indicating the most likely genotype at the corresponding position. More precisely, given an input variant *v*, the k-mers covering $b_{ref}$ ($b_{alt}$) are called reference (alternative) *signatures*. The prediction is usually driven by assumptions concerning the different distributions of the signatures generated respectively by the homozygous and the heterozygous variants.

The first system to implement an AF approach for SNP genotyping is LAVA [3]. It uses k-mers to connect variants to their positions in reference and alternative genomes. Then, it uses a dictionary for heuristic mapping of reads, enabling mismatch detection and coverage assessment for SNP candidates by examining read alignments near SNP locations. Genotype predictions are made using Bayes’ theorem, based on coverage data.

*Vargeno* [4] improves LAVA by providing a faster and more accurate solution for *SNPs* genotyping by using optimized k-mer dictionary queries and improved mapping quality control.

Despite their excellent performance, Vargeno and LAVA are very memory-demanding and require almost a terabyte to complete the analyses on a single individual. MALVA [5] proposes a more space-efficient algorithm. By analyzing k-mer histograms from input reads, it distinguishes between homozygous and heterozygous variants without mapping reads, applying LAVA’s statistical framework for genotyping SNPs, multiallelic polymorphisms, and small indels, even if at a slower pace and potentially lower accuracy due to non-unique k-mer signatures.

Nebula [6] is another AF framework that focuses on genotyping known structural variants by considering only non-ambiguous alternative signatures, leveraging pre-estimated likelihood functions from known genotypes, thus bypassing the need for direct reference-to-alternative comparisons.

PanGenie [7], the latest AF framework, incorporates a pangenome graph for a more accurate representation of species variability, although its application is limited by slow runtime and higher memory requirements.

#### Reference-free AF algorithms

These algorithms identify differences between individuals without requiring prior knowledge of the entire genome of the species. Popular methods like, e.g., [8–11] build a *De Bruijn* (DB) graph, starting from the k-mers extracted from the input reads.

The vertices of a DB graph represent the distinct k-mers found in the genomic reads used to create the graph. The edges connect vertices corresponding to adjacent k-mers, i.e., k-mers that overlap for $k-1$ characters. Edge directions suggest how the k-mers corresponding to two adjacent vertices can be merged to assemble the genome of a species. Variants between sequenced individuals lead to the creation of multiple *branching paths* describing the same parts of the genome. In particular, the presence of an isolated *SNP* generates a *simple bubble* subgraph consisting in two parallel paths sharing the starting and the ending vertex.

Some applications of DB graphs in variants calling are described in [8, 9]. In these two articles, graph coloring techniques are used to detect polymorphisms between individuals. The former is only able to assess the presence of variants, while the latter also classifies polymorphisms as homozygous or heterozygous.

DB graphs tend to become very large and, consequently, difficult to store, when based on mammalian-sized datasets. A possible solution to this problem consists of introducing compressed data structures for storing a *De Bruijn* graph. This issue is addressed in [10, 11] with a compact *De Bruijn* graph representation using string compression and indexing, like the positional Burrows-Wheeler transform [12].

Further solutions take into account a probabilistic representation of the *De Bruijn* graph. Among the proposed techniques, *DiscoSnp* [13] is one that outperforms many others in terms of space and time efficiency, when used to retrieve isolated *SNP* on genomes of complex organisms. It features an efficient storage layout, where only the vertices of an input *De Bruijn* graph are stored using a cascade of Bloom Filters. The edges are found by properly querying these filters to obtain paths corresponding to isolated *SNPs*, even though the Bloom Filters may return non existing paths due to false positive matches.

Two reformulations of this algorithm are implemented using the library *GATB* [14]. *DiscoSnp++ *[15] tries to detect any kind of *SNP* and indels with a small size, while *DiscoSnpRad* [16] is the adaptation of *DiscoSnp* for *RAD* sequencing data.

We notice, however, that the usage of compression techniques, while allowing to analyze dataset that would rather be untractable because of memory problems, tends to negatively influence the time performances of the different variants calling pipelines proposed so far.

### The MapReduce distributed computing paradigm

Computer clusters enable the processing of huge collections of data through distributed computing architectures. These systems connect a number of computers (called *nodes*) to work together for solving a single problem. The nodes coordinate the usage of their computing and memory resources using messages exchanged over a network.

The *MapReduce* (MR) paradigm [17] is a programming model designed for implementing distributed algorithms. It considers an input dataset of key-value pairs distributed across a computer cluster and provides a flow that combines two types of functions:

- *map functions*. They process each key-value pair to obtain a new (possibly empty) set of key-value pairs.
- *reduce functions*. They first collect on a same node all pairs sharing the same key and, then, they aggregate all values in this collection, returning a (possibly empty) set of key-value pairs

*MapReduce* algorithms are usually organized in complex pipelines, where the outputs of map or reduce functions are used as inputs for the next function. Map and reduce functions are executed, as *tasks*, by the computing units available on the nodes of the computer cluster. The degree of parallelism depends on the number of independent tasks that can be run in parallel on an input collection of key-value pairs. The execution of reduce functions may have a very negative impact on the time performance of a *MapReduce* algorithm, because of the time to be spent for moving key-value pairs from their original nodes to their destination nodes, for aggregation.

#### Apache spark

*Apache Spark* [18] is the standard framework for developing *MapReduce* algorithms to be executed on a distributed computing architecture. On this type of architecture, Spark organizes iterative procedures that involve multiple parallel operations on a set of data partitioned across nodes. The units of work are scheduled according to a task dependency diagram.

*Resilient Distributed Dataset* are the core data structures of Spark. RDDs are an abstract representation of key-value pairs distributed across the computing cluster. These distributed collections are processed by operations (called *actions* and *transformations*) that implement MR functions on a Spark cluster. The contents of an RDD can be retrieved locally to return the output of the distributed operations.

*Apache Spark* also provides libraries (built on top of RDDs) dedicated to specific tasks. The *GraphX* API [19] is designed for large-scale graph processing on a Spark cluster [20].

This API introduces a distributed representation of a graph built on top of two RDDs, storing respectively the vertices and the edges of a graph, with their associated properties. In details, each vertex is associated with an automatically-generated unique id (*vid*) and (optionally) a set user-defined attributes. The edges are modeled as triplets containing the source vertex id, the destination vertex id, a virtual partition identifier (*pid*) and (optionally) a set user-defined attributes.

Vertices and edges are scattered across the nodes of a computer cluster, according to their vid/pid. The GraphX API employs a default partition strategy and generates the so-called *VertexMap* RDD that maps each vertex to the list of partitions containing its incident edges. Leveraging on the vertices and the edges identifiers, GraphX allows also to consider user-defined graph partition strategies that may minimize the traffic data and balance the workload of the compute nodes.

*GraphX* allows to traverse and to analyze a graph, by developing a distributed algorithm implemented in Pregel [21]. It is a computational model proposed by Google for large-scale graph processing and based an iterative approach. In each iteration of a Pregel excecution, vertices of a distributed graph interact with their adjacent ones by sending and receiving messages. Upon reception of a new message, a vertex can modify its internal state, by processing the content of the received message by means of a user-supplied function.

The propagation of messages takes places only through *active* triplets, that are, triplets satisfying given conditions on their attributes. It stops when there are no more active triplets. The workflow is divided into a series of *supersteps*, each made of three steps:

- *Step 1*. The two vertices of each active triplets share information about their attributes by means of messages.
- *Step 2*. Messages addressed to the same vertex, but exchanged in different triplets, are merged to produce a single overall update.
- *Step 3*. Updated attributes may lead to the activation or deactivation of triplets, thus triggering or not the execution of another superstep.

*Step 1* and *Step 3* are implemented by *GraphX* using *map* functions, while *Step 2* requires a *reduce* function.

## Methods

### Motivations

Algorithms for isolated SNPs calling using *De Bruijn* graphs are generally efficient but very memory hungry or, when using compressed data structures, require long execution times. These limitations may prevent their use in the analysis of very large and complex genomic datasets.

In this work, we propose a novel isolated SNPs calling pipeline based on distributed *De Bruijn* graphs. Our approach aims to overcome the memory and runtime limitations of traditional algorithms by properly exploiting the available resources of a distributed system.

### Our proposal

Our approach is based on the MR paradigm (see “The MapReduce distributed computing paradigm” section) and exploits the resources of a distributed computing architecture. This addresses the problem of the enormous memory required to store the entire *De Bruijn* graph, without resorting to compressed or probabilistic data structure [15, 22].

**Fig. 1:**
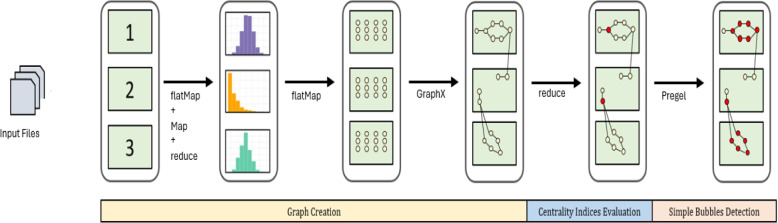
The workflow of the distributed approach for isolated *SNPs* calling. The input reads are loaded into memory and scattered across different partitions (three in this example) of a distributed data structure. Then, the frequencies of the $(k+1)$-mers are computed to filter out the low-covered ones. From the $(k+1)$-mers the vertices are extracted and a distributed *De Bruijn* graph is initialized. By computing the degrees of the vertices, the starting vertices of the bubbles are detected. Finally, a Pregel algorithm finds all the simple bubbles in the *De Bruijn* graph

Our pipeline is formulated as a sequence of distributed transformations, starting from an initial distributed collection $\mathcal {C}$ of reads. Figure 1 summarizes the workflow of the proposed approach.

**Fig. 2:**
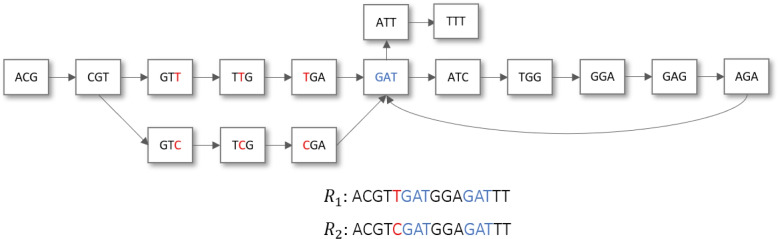
The *De Bruijn* graph obtained from two sequenced reads ($R_{1}$ and $R_{2}$), when $k=3$. The set of vertices represent all the distinct 3-mers extracted from $R_{1}$ and $R_{2}$, while the edges connect the overlapping 3-mers. The *SNP* (marked in red) between $R_{1}$ and $R_{2}$ generates two parallel paths connecting CGT with GAT. The substring GAT occurs twice, determining a loop in the graph and two branching paths

Initially, a DB graph $\mathcal {G}$ is created starting from all reads in $\mathcal {C}$. Then, $\mathcal {G}$ is processed by a distributed algorithm that identifies all simple bubbles, i. e., the divergent paths arising due to presence of a genomic variant (see Fig. 2). Finally, all isolated bubbles, i.e., the *SNPs*, are returned as output.

Notice that our pipeline can be easily extended to handle the case where there are multiple collections of input reads, each leading to a different *De Bruijn* graph. More details on the pipeline follow.

#### Graph creation

This step aims to build a distributed *De Bruijn* graph of k-mers occurring in the input samples. Given a pair of FASTA/FASTQ files, each containing a collection of reads extracted from another sample, their contents are loaded and held in memory using two distributed collections of strings. Reads are loaded using the FASTdoop library [23].

Then, all $(k + 1)$-mers are extracted from each read of the two samples and stored in two distributed data structures encoded as long numbers, each associated with frequency 1. This is done using a distributed flatMapToPair transformation.

Then, a distributed reduce transformation is used to count the total frequency of each $(k + 1)$-mer by summing all its occurrences. The result is encoded as an RDD containing a distributed collection of $(k + 1)$-mers with their associated frequencies (coverage), one per sample.

At this point, the contents of the two RDDs containing the frequencies of all $(k + 1)$-mers are processed using a distributed mapToPair transformation. This transformation filters out all $(k + 1)$-mers with coverage lower than a threshold $\tau$, as these are assumed to be due to sequencing errors

Before graph construction, all samples are merged. Now, each $(k + 1)$-mer can be considered as one edge of a DB graph connecting the vertices associated to the two corresponding k-mers. So, we first create a new DB graph $\mathcal {G}$ as a GraphX object. Then, we insert a set of vertices into $\mathcal {G}$ by extracting the two consecutive k-mers existing in all previously found $(k + 1)$-mers. Finally, for each $(k + 1)$-mer entered, we connect the vertices associated to the corresponding k-mers adding a new direct edge in $\mathcal {G}$. The same is done with the backward strand of each $(k+1)$-mer.

We recall that a GraphX graph is represented by a set of triplets, each containing an edge and its corresponding source and destination vertices.

#### Centrality indices evaluation

This step computes two centrality indices, reporting the number of incoming and outcoming edges for each vertex *v*, respectively denoted *inDegree* and *outDegree*.

For each vertex *v*, this is done by counting the number of triples containing *v* in $\mathcal {G}$, distinguishing the cases where *v* is the source vertex from those where it is the destination vertex. Then, for each vertex, these counts are aggregated by a distributed reduce transformation, to obtain the corresponding centrality indices.

#### Simple bubbles detection

The simple bubbles detection algorithm use a Pregel algorithm where *path-building* messages are distributed over the graph to discover all paths describing this type of bubbles.

Initially, we consider vertices from which at least two distinct paths originate, i.e., those with an *outDegree* greater than 1. We refer to these vertices as “starting vertices”. Then, all triplets in $\mathcal {G}$ that contain a starting vertex are marked as enabled (i.e., the *starting points*).

Then, each activated triplet sends a message to its neighbors along the outcoming edges, containing information about the current path (which initially contains only the starting point) and the iteration number. We define this as the first iteration of the detection algorithm.

In iteration *k*, triplets with vertices that received a message in the previous iteration are activated. When a vertex receives a *path-building* message from one of its neighbors, it updates the received message by adding its own identity and increases the iteration number. The iteration number is also stored by the vertex in its state. Finally, the vertex sends the updated message to its neighbors along the outgoing edges.

To deal with branching bubbles, for the first *k*−1 iterations, the message *path-building* is transmitted only if the destination vertex has one incoming edge and one outcoming edge. At iteration *k*, *path-building* messages are sent without checking the degrees of the destination vertex. This is because the destination vertex at iteration *k* should be the ending vertex of the bubble and, in this case, more than one incoming edge and more than one outgoing edge are allowed.

After *k* iterations, the algorithm identifies vertices with iteration number $k + 1$ that received two messages with paths of equal length starting from the same vertex.

These vertices are the terminal vertices of sequence pairs that contain simple bubbles and thus report isolated SNPs. They are encoded as a distributed collection of string pairs.

### Cluster-driven partitioning strategy

A distributed approach allows our pipeline to bypass the memory constraints of many traditional SNPs calling applications. However, although it is true that distributed system may provide a virtually unlimited amount of memory, it is true as well that a bad usage of these resources can lead to very unsatisfying time performance.

This is particularly relevant for graphs, where a bad mapping of their structure onto a distributed system can drastically increase the cost of even very simple operations like, e.g., exploring the neighborhood of a vertex. This happens because the data involved in a traversal operation may not be available on a single node but scattered over several nodes. In the worst case scenario, for each edge to be crossed along a path, the end vertex resides on a different vertex than the origin vertex (we call these *x-cross* paths).

We recall from “Apache spark” section that the GraphX API used by Spark for graph processing includes a naive partitioning strategy, used to create a distributed representation of an input graph. However, by design, this strategy only uniform distribution of graph components across compute nodes. Instead, it lacks optimizations for ensuring data locality during the exploration of vertices and their connections.

We expect an optimized partitioning strategy to significantly reduce, when not eliminating at all, the number of *x-cross* paths. This could significantly reduce the communication overhead between different compute nodes and/or computing units, thus shortening execution times.

To test this idea, we introduce a partitioning strategy based on a hierarchical clustering approach to group reads referring to the same genomic region. Then, assuming all elements of a cluster are processed on a same compute node, this would enable to perform bubbles detection locally on each node.

Our strategy aims to improve the partitioning of a *De Bruijn* graph in a distributed system for the particular case of bubbles detection. It is based on the assumption that clustering the reads according to an appropriate similarity metric isolates specific genomic regions. Consequently, assigning a cluster to one node allows for efficient bubble detection with minimal communication overhead, enhancing performance and scalability. The proposed strategy consists of two steps:

- Step 1: Hierarchical Clustering: the collection of reads from two individuals are clustered using a bottom-up approach and a greedy stop criterion.
- Step 2: Clusters Binning: the clusters returned in the previous step are grouped into *B* distinct bins across the distributed system. The generation of the bins is driven by the application of the longest processing time first rule (i.e., *LPT* rule) [24], to ensure for a uniformly distributed workload.

#### Hierarchical clustering

Our strategy efficiently groups reads from two individuals into clusters likely covering the same genomic region. We achieve this using a bottom-up hierarchical clustering algorithm.

The algorithm implements an iterative procedure that progressively merges similar elements into larger clusters to eventually obtain a *dendrogram*, i.e., a hierarchical tree-like structure representing the clustering relationships. In the initialization step, each read is considered as a single-element cluster. Then, in the *i*-th iteration and for each cluster, the read closest to the others in the same cluster is selected as its *representative*. Then, the pairwise similarity matrix between all clusters is calculated and the two closest clusters are merged into one. This process continues until a single cluster remains.

For very large datasets, building the entire dendrogram can be very computationally intensive. Moreover, we prefer having more clusters, but consisting of very similar reads referring to the same genomic region, rather than having few cluster with distant reads.

To achieve this, we incorporated a greedy stopping criterion into the algorithm. Namely, the merging process stops when the similarity between the two closest clusters is below a threshold *c*. This avoids the need for manual evaluation and automatically determines the number of clusters.

However, choosing the right value of *c* is crucial. A high value of this parameter leads to many small clusters while a low one might increase the chance of allocating over different compute nodes vertices contributing to a bubble.

#### Choice of a similarity measure

We recall that a genomic read is simply a string of characters. Thus, our clustering framework needs a similarity measure that can capture the underlying similarity patterns between strings and is not extremely sensitive to slight variations in characters. This helps to ensure that reads sequenced from different individuals but covering the same genomic regions are clustered together, despite the presence of *SNPs* in the same cluster.

In this work, we consider the *Jaccard index*, also known as *Jaccard similarity coefficient*. This measure is widely used in computational genomics to assess similarity between nucleotide strings and for clustering [25, 26]. The Jaccard index quantifies the similarity between two reads with respect to their shared k-mers. To calculate it, we extract the sets of k-mers from each read and determine the percentage of k-mers they share. Mathematically, the Jaccard index can be expressed as follows:

$$\begin{aligned} J(A, B) = \frac{{|A \cap B|}}{{|A \cup B|}} \end{aligned}$$

where:

- *A* and *B* represent the sets of k-mers extracted from the two reads;
- |*A*| and |*B*| represent, respectively, the number of distinct k-mers in *A* and *B*;
- $|A \cap B|$ represents the number of shared k-mers between *A* and *B*;
- $|A \cup B|$ represents the total number of unique k-mers in the union between *A* and *B*.

The Jaccard index ranges between 0 and 1. The more it is larger, the more the reads are similar. When it is equal to 1, the reads are identical. Since the Jaccard index evaluates the presence or the absence of shared k-mers, it is robust to small variations in the reads being compared.

##### Binning the clusters

The clusters returned in the previous step need to be distributed among the computational units of the distributed system to perform bubble detection in parallel. However, uneven read distribution and highly repetitive genomic regions can lead to the creation of overloaded clusters. Therefore, the adoption of an appropriate scheduling model is needed to ensure for a balanced workload across the computational units.

We consider this as a scheduling problem with identical parallel machines and no preemption. The objective is to minimize the maximum completion time (makespan) by assigning each of the *n* jobs, characterized by their respective processing times $\{p_j$,$j = 1,\ldots , n\}$, to one of the *m* parallel identical machines. In our context, jobs correspond to the tasks associated with building and exploring the local DB graph belonging to a cluster of reads. Processing time of each job is estimated based on the number of k-mers extracted from the reads within the same cluster. The machines, on the other hand, represent the available computational units. This problem, referred to as $P||C_{max}$, is known to be NP-hard and can be formulated mathematically as follows:

1 $$\begin{aligned} & \textrm{min}\ C_{max}\end{aligned}$$

2 $$\begin{aligned} & C_{max} \ge \sum\nolimits_{j=1}^N p_j x_{ij} \quad \forall i \in \{1..m\}\end{aligned}$$

3 $$\begin{aligned} & \sum\nolimits_{i=1}^m x_{ij}=1 \quad \forall j \in \{1..N\}\end{aligned}$$

4 $$\begin{aligned} & x_{ij} \in \{0,1\} \quad \forall i \in \{1..m\} \,\, j \in \{1..N\} \end{aligned}$$

where $C_{max}$ is the *makespan*, *N* is the number of jobs, *m* is the number of machines, $p_j$ is the processing time of job *j*, $x_{ij}$ is the decision variable. The value is 1 if the job *j* is assigned to machine *i*, otherwise it is 0. Given the complexity of this problem, many heuristic solutions have been proposed in literature, as the *LPT* rule. It involves sorting jobs in a non-increasing order based on their processing times and assigning each job iteratively to the machine that currently has the minimum completion time. The completion time of a machine is determined by summing the processing times of all the jobs assigned to that particular machine.

## Results

We conducted an experimental analysis for evaluating the performance of our pipeline, both in terms of its scalability and of the quality of its output.We also evaluated its performance with respect to DiscoSNP [13, 15], a state-of-the-art genotyping tool. In the following, we provide details about our experimental setting and the outcomes of the proposed experiments.

### Datasets

We consider the alignments of two human individuals, (*HG002* and *HG003*), provided by the GIAB consortium [27]. Then, we use SAMtools to extract the reads corresponding to chromosomes 2, 7 and 22. These datasets will be referred to as *Chr2*, *Chr7*, and *Chr22*, respectively, with sizes of 19 GB, 12 GB, and 5 GB.

The chosen genomes are also provided with a VCF file containing highly confident variants with respect to the chromosome primary assembly (*G*). We filter from this the set of isolated *SNPs* to generate the sequences $G_{2}$ and $G_{3}$. $G_{2}$ and $G_{3}$ are obtained by inserting in *G* the *SNPs* targeting, respectively, HG002 and HG003. Both these sequences are used to find the polymorphisms differentiating the two individuals and assess the scalability of our pipeline by mapping back the detected bubbles.

### Computing environment

All our tests have been performed on an HPC infrastructure equipped with 8 compute nodes running Linux, each equipped with 2 AMD Epyc 7452 processors, 64 compute cores and 256 GB RAM, for a total of 512 compute cores (see [28] for more details). We used version 3.1.3 of Apache Spark and version 2.7 of Apache Hadoop.

### Assessing the accuracy

We evaluated the output accuracy of our pipeline by evaluating the precision and the recall rates on the *Chr7* dataset, as a function of *k* and $\tau$. In our case, the precision rate is the percentage of true bubbles returned by our pipeline, while the recall rate is the percentage of bubbles in $\mathcal {B}$ detected by our pipeline.

*Sensitivity to* *k*. The results, available in Table 1, show that our pipeline yields high-quality results (with precision and recall consistently above $90\%$) for large values of *k* (i.e., 27 and 31). We also note that bubble detection is significantly affected by the size of the k-mers. As *k* decreases, erroneous k-mers become harder to filter out, since the cardinality of the k-mers set decreases, determining an increase of the average k-mers frequencies. At the same time, this determines a growth of branching bubbles that our pipeline does not cover, leading to poor performance in terms of recall rate. However, it should be also noted that, when $k=31$, a slight decrease in the recall is observed due to the smaller number of k-mers whose frequency is larger than $\tau$.

*Sensitivity to* $\tau$. Table 2 reports how precision and recall change are influenced by $\tau$. The results show that recall decreases as $\tau$ increases, likely because of low covered *SNPs*. This likely affects heterozygous *SNP* detection, as these polymorphisms, targeting a single allele, have lower coverage than homozygous ones. On the other side, when $\tau$ is smaller than 10, we notice a drastic degradation of precision and recall. Further analysis show that this degradation is due to the growth of erroneous paths and branching bubbles that are not covered by our pipeline.

**Table 1:** Accuracy results of our pipeline for different values of *k*, using $\tau =12$

k	Precision	Recall
11	$17.50\%$	$0.08\%$
15	$69.75\%$	$9.33\%$
19	$83.32\%$	$62.01\%$
23	$92.51\%$	$81.09\%$
27	$96.92\%$	$96.82\%$
31	$97.16\%$	$95.24\%$

**Table 2:** Accuracy results of our pipeline for different values of $\tau$, using $k=31$

$\tau$	Precision	Recall
8	$29.03\%$	$41.17\%$
10	$84.65\%$	$85.28\%$
12	$97.16\%$	$95.24\%$
14	$96.01\%$	$79.51\%$
16	$97.54\%$	$52.15\%$
18	$97.08\%$	$29.11\%$

### Scalability

When using a distributed approach, one expects that the solution time of a given problem could be reduced by just employing more computational resources. However, the ability to efficiently exploit the computational capability of a distributed system is not always a given. This is either due to the inherent non-parallelism of the problem being solved or due to poor algorithmic design and implementation.

We addressed this problem by conducting an experiment to measure the scalability of our pipeline while processing the *Chr7* dataset, as a function of the number of computing units used, ranging from 24 to 96.

**Fig. 3:**
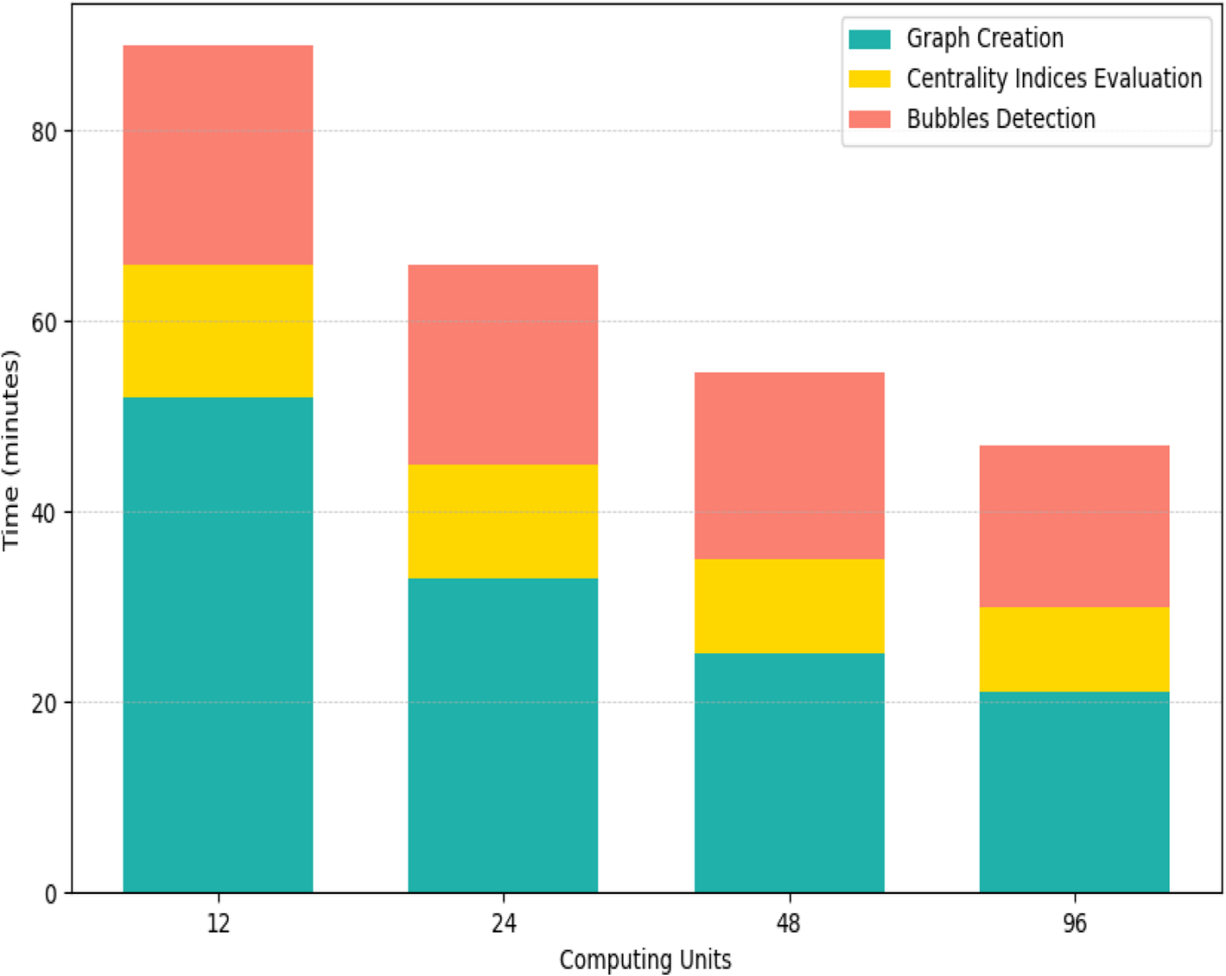
Execution times, in minutes, required by our pipeline for isolated *SNPs* calling on the Chr7 dataset, using an increasing number of computing units

As can be seen in Fig. 3, our pipeline succeeds in exploiting an increasing number of computing units, which is reflected in the decreasing overall execution time. In details, it scales well when using up to 48 computing units, especially during graph creation. Instead, switching to 96 computing unites does not yield the performance gain one would expect. Closer inspection reveals that, in this setting, the scalability of our pipeline is less pronounced because of the sub-optimal partitioning strategy employed by GraphX to represent the distributed *De Bruijn* graph.

**Fig. 4:**
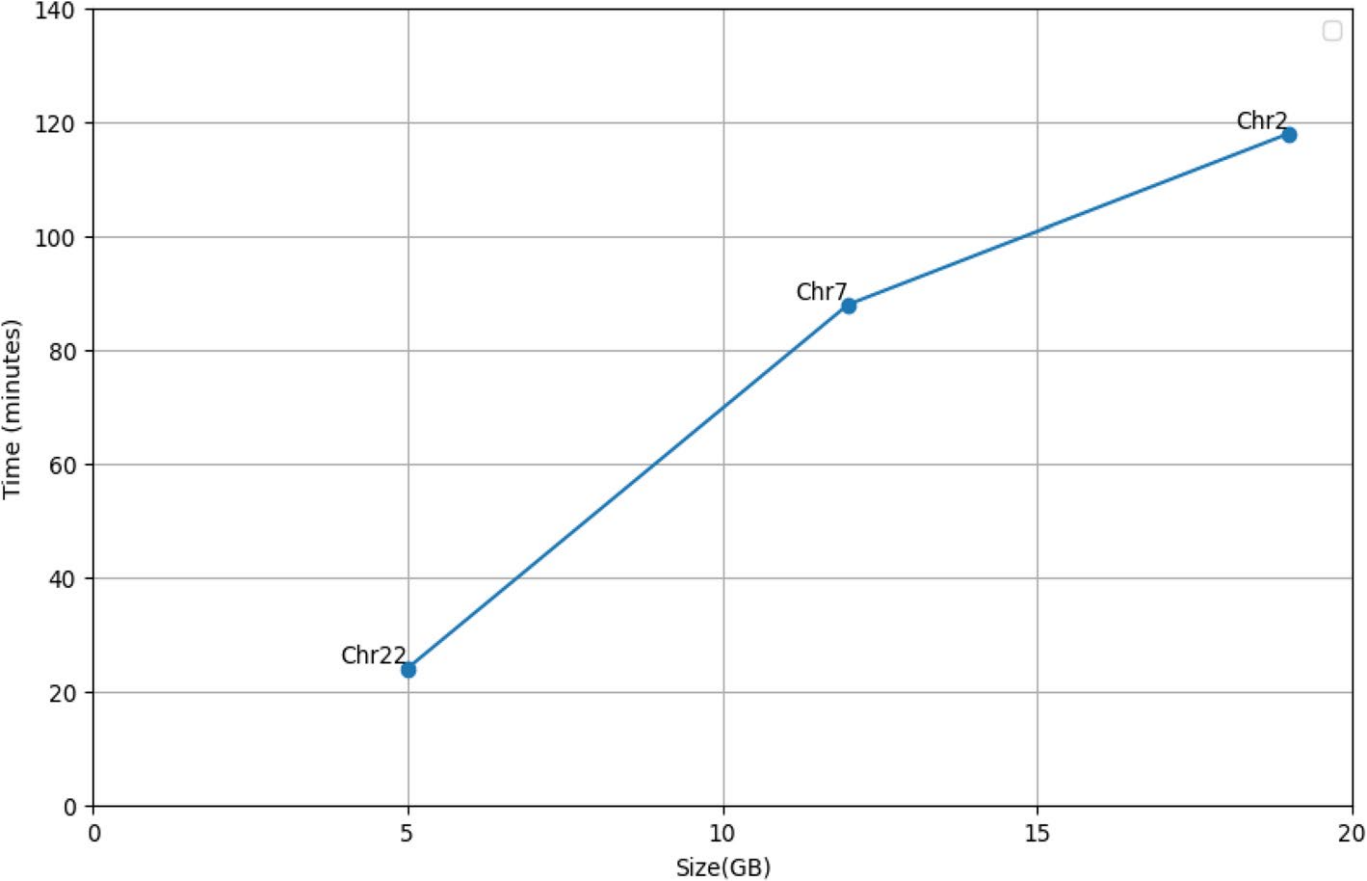
Execution times, in minutes, required by our pipeline for isolated *SNPs* calling, increasing gradually the size of the dataset

We conducted a further experiment to assess the scalability of our pipeline with respect to the input dataset size. Namely, we compared the time-performance of our pipeline when executed on each of three different datasets considered in our study. The results, available in Fig. 4, show that the execution time of our pipeline grows faster than the input dataset size. At a closer inspection, it turns out that this slow-down is due to the preliminary task where k-mers are extracted from input sequences and aggregated (more details about this are given in “Comparative analysis” section).

### Comparative analysis

**Fig. 5:**
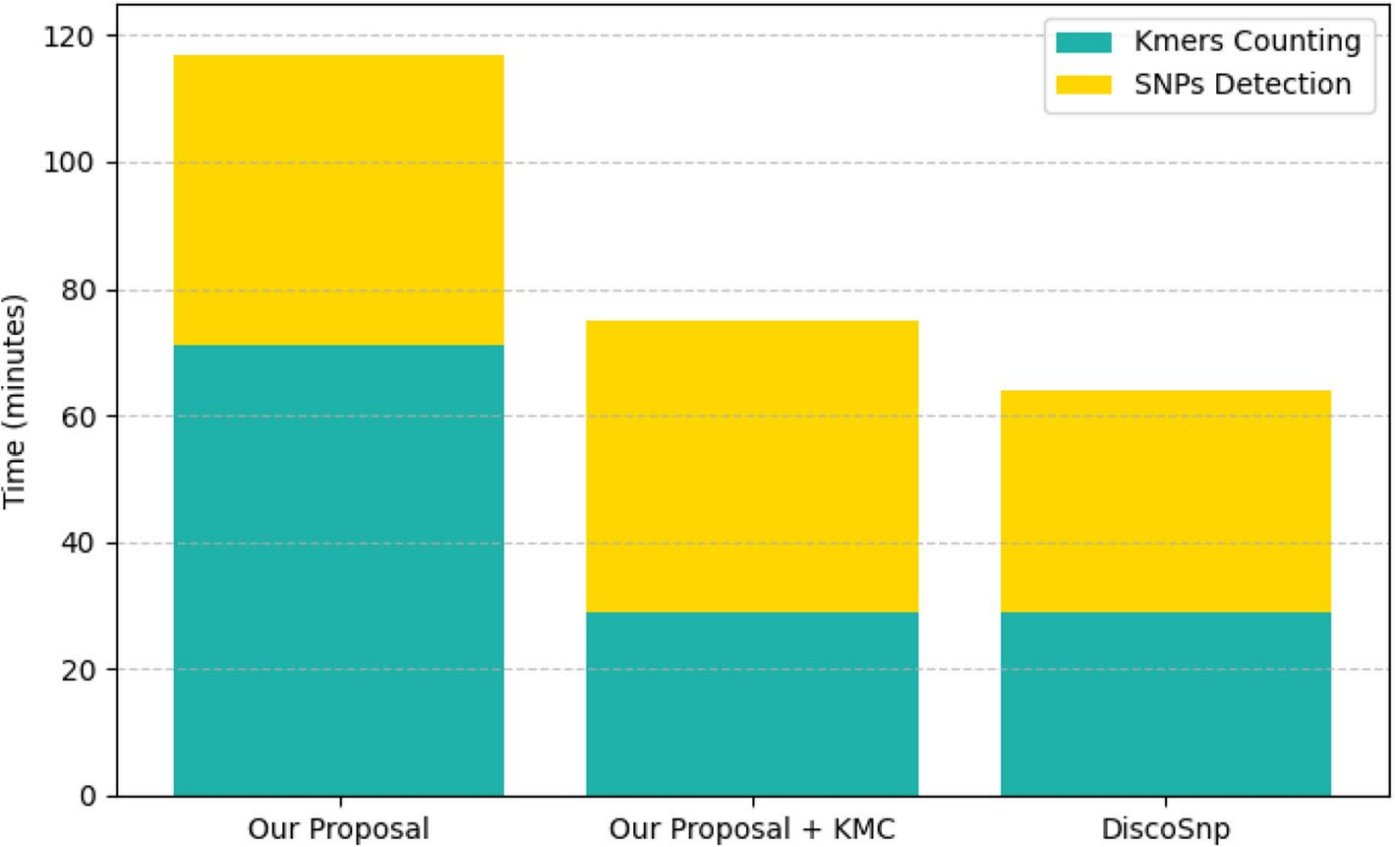
Execution times, in minutes, required by our pipeline and *DiscoSnp* to process the *Chr2* dataset

In this experiment, we compared the time-performance of our pipeline with *DiscoSnp*, a state-of-the-art genotyping tool. To this end, we measured the time required to process the *Chr2* dataset (i.e. the largest among the ones we considered) using either of the two tools. The results, presented in Fig. 5, show that *DiscoSnp* easily outperforms our pipeline in this setting, while using the same number of computing units. Thus, we investigated the reasons behind such a performance gap and found out that the preliminary task executed by our pipeline for the extraction of the k-mers from the input sequences and their later aggregation, is much slower than the equivalent *DiscoSnp* task. As for *DiscoSnp*, this task is not fulfilled internally but it is delegated to an external tool that is specialized for this purpose: KMC [29]. Starting from this observation, we developed an alternative version of our code using KMC as well to perform the initial k-mer count task, so as to make the comparison with *DiscoSnp* more fair. Thanks to this modification, the time-performance of our pipeline is now much closer to *DiscoSnp*.

### Assessing graph partition strategy

Starting from the *Chr*7 dataset, we selected uniformly at random 10, 000 bubbles and extracted the corresponding *De Bruijn* graph using, respectively, the GraphX default partitioning strategy and our cluster-driven one. From now on, we refer to the filtered *De Bruijn* graph used in our analysis as DB1e4.

**Fig. 6:**
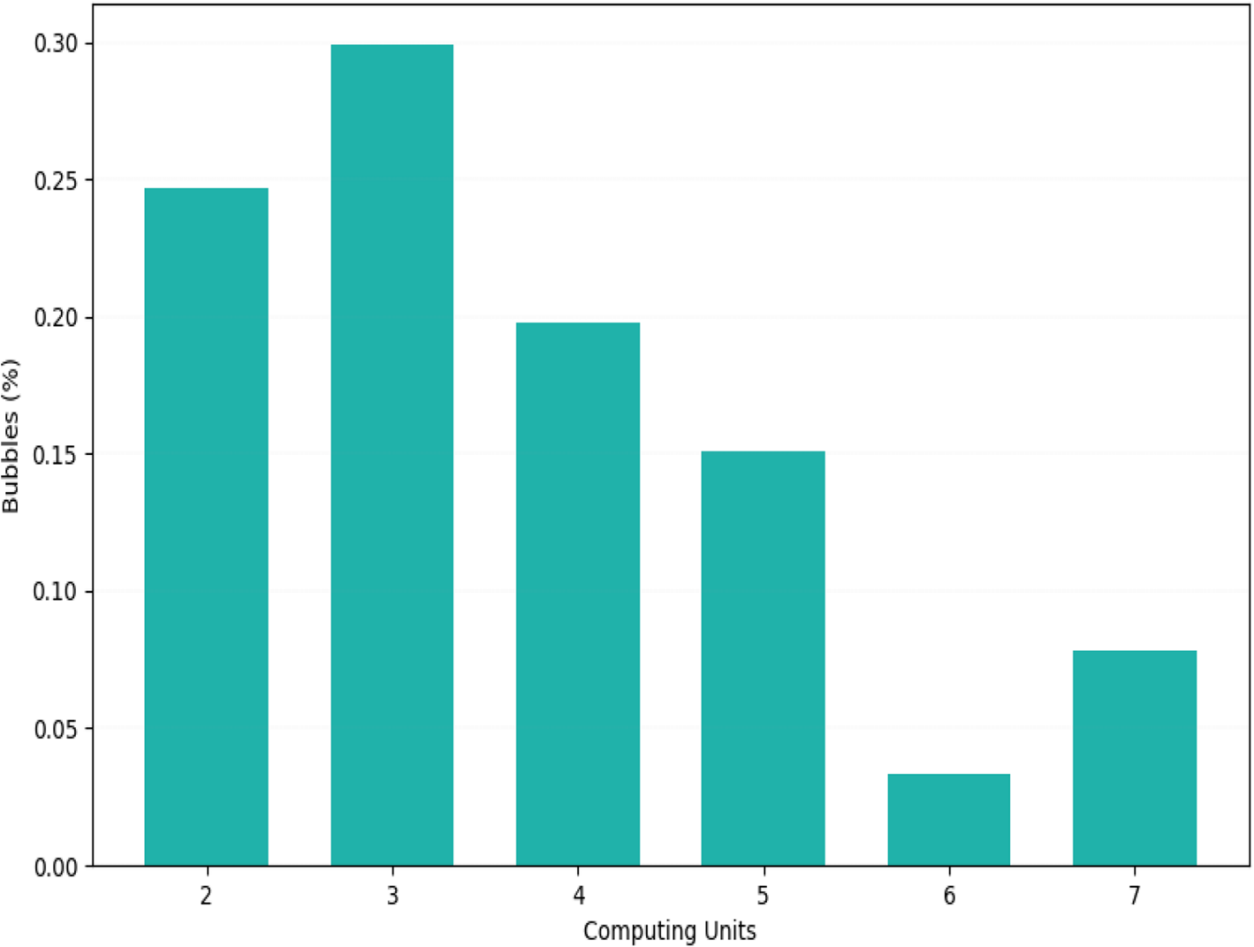
The histogram illustrates the distribution of the bubbles, in terms of the number (in percentage) of computing units to involve for traversing them, according to the default GraphX partitioning strategy

Then, we determined the number of computing units needed to traverse each bubble (up to 31 units due to k=31). The results show that no bubble is traversable within a single unit (see histogram in Fig. 6). Many require at least two units, with the most frequent case requiring the involvement of three computing units.

**Fig. 7:**
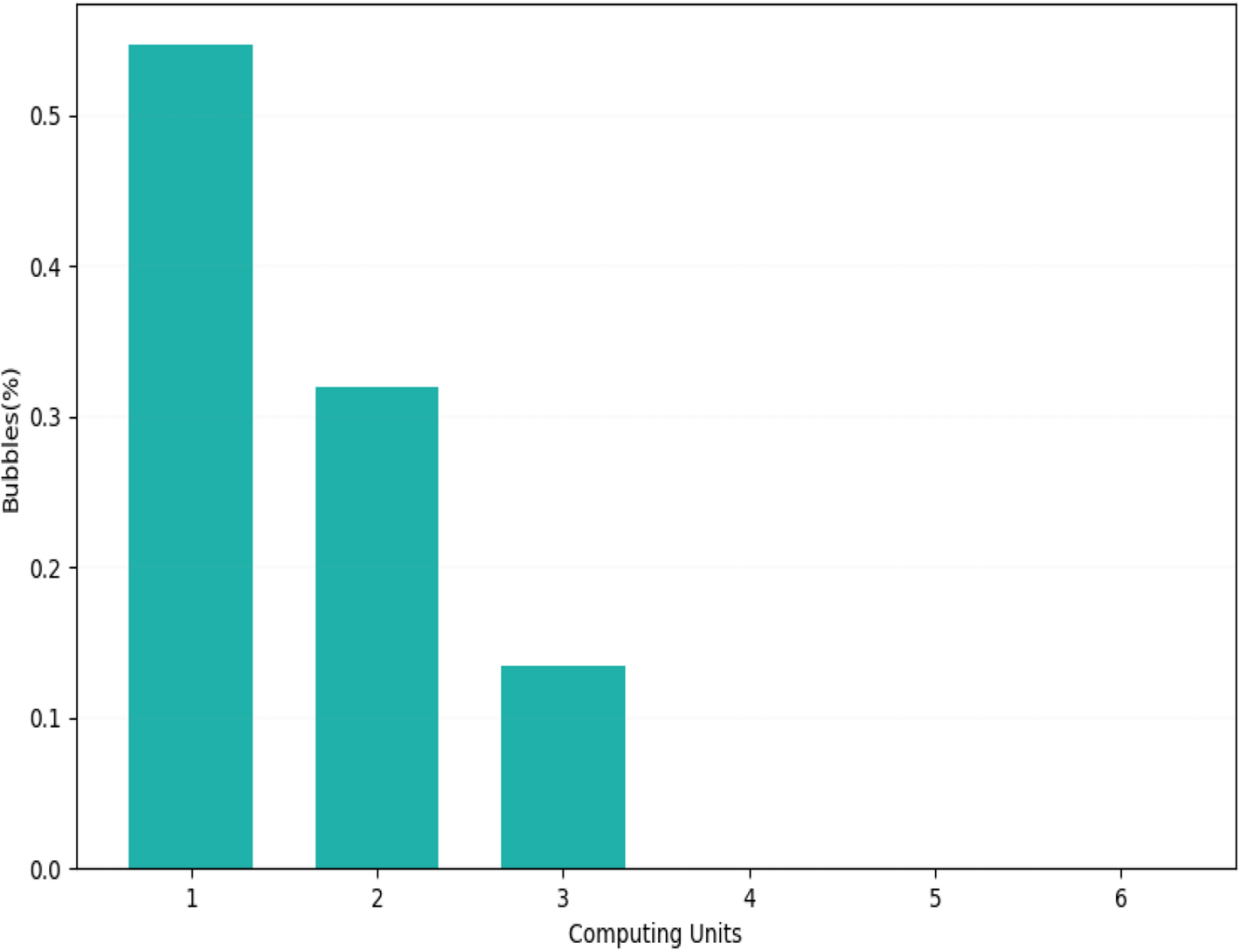
The histogram illustrates the distribution of the bubbles, in terms of the number (in percentage) of computing units to involve for traversing them, according to the cluster-driven partitioning strategy

We evaluated the impact of our cluster-driven partitioning strategy on the scalability of our pipeline, with respect to the default partitioning strategy employed by GraphX. The results, available in Fig. 7, show that the adoption of our partitioning strategy leads to a significant drop in the number of cross-node paths.

Next, we ran the *SNPs* calling procedure on DB1e4 with the default partitioning and increasing computing units. Then, we repeated the same experiment with our hierarchical clustering and binning scheme, using a number of bins that was 4 times the number of allocated computing units.

**Fig. 8:**
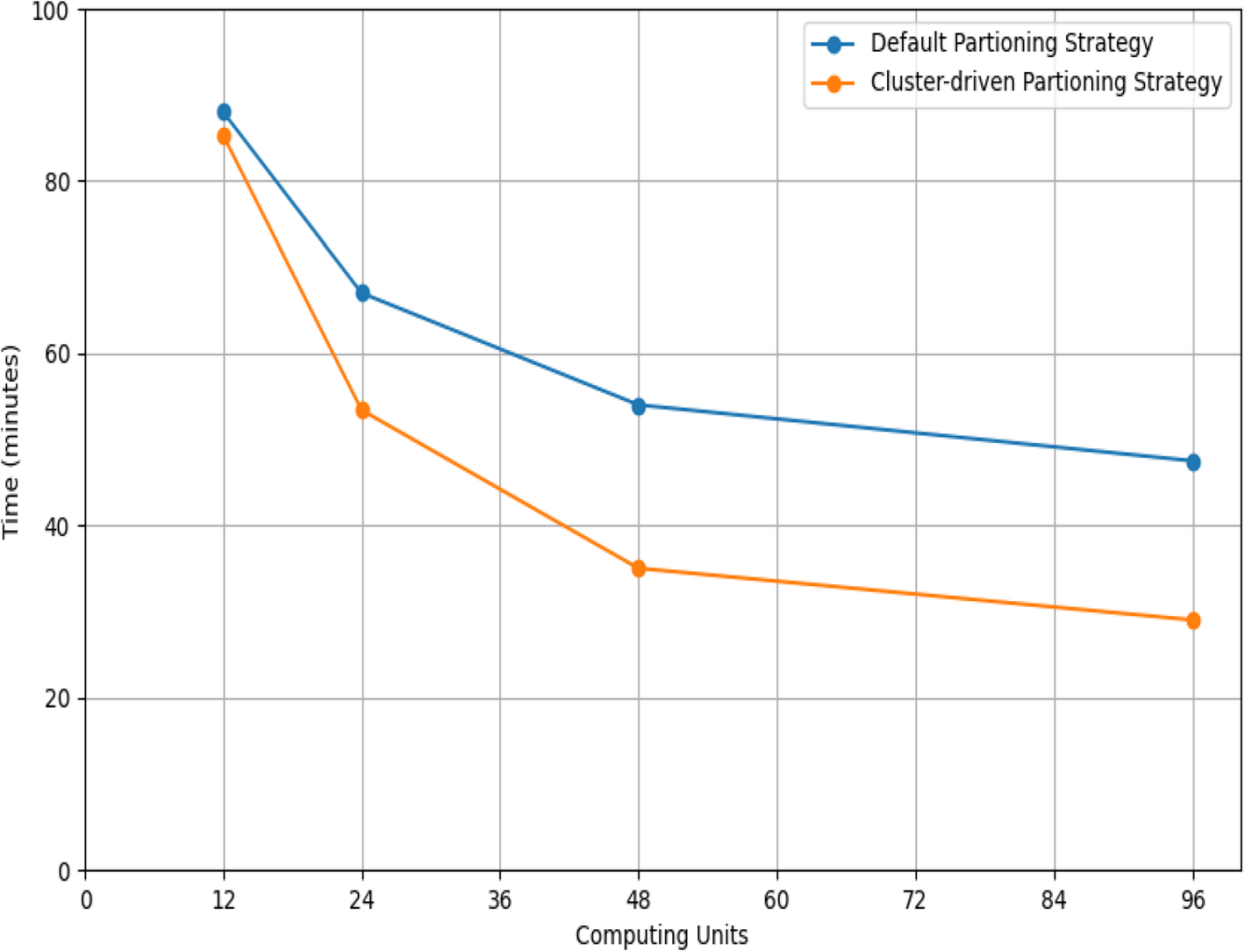
Time performances of the *SNPs* calling procedure executed on the DB1e4 graph, when considering the GraphX default partitioning and the cluster-driven partitioning strategies. The execution times are normalized and reported as a function of the employed computing units

Figure 8 compares the scalability deriving from the application of the two partitioning approaches, highlighting how our proposed strategy significantly improves the standard partitioning strategy on an increasing number of computing units.

## Discussion

In this work, we addressed the challenge of large-scale isolated *SNPs* calling by introducing the first reference-free distributed genotyping pipeline for detecting isolated *SNPs* based on *De Bruijn* graphs. The experimental results demonstrated a remarkable output quality, but also revealed a performance bottleneck in the pipeline, primarily due to the standard graph partitioning strategy used by GraphX. To address this issue, we developed an optimized partitioning approach based on hierarchical clustering and Jaccard similarity, which improved the efficiency and scalability of our pipeline.

## Conclusions

In conclusion, while the optimized partitioning strategy has enhanced the scalability of our pipeline, it also introduced a potential drawback: excessive overhead. This overhead may offset the advantages gained in terms of efficiency, particularly in very large datasets. As a result, we are currently exploring more advanced partitioning techniques beyond hierarchical clustering, aiming to reduce this overhead and further optimize the scalability of our approach. Additionally, we plan to compare the performance of our pipeline with state-of-the-art non-distributed genotyping tools and extend it to cover a wider range of genomic variations, such as non-isolated *SNPs* and indels, while developing a robust statistical framework for accurate genotype prediction and quality assessment.

## Data Availability

The data used in this study are publicly available through the *Genome in a Bottle Consortium*. They can be accessed and downloaded directly from the official GitHub repository at: https://github.com/genome-in-a-bottle.

## Abbreviations

AF: Alignment-free
NGS: Next generation sequencing
SNP: Single Nucleotide Polymorphism

## Footnotes

1 Throughout all the paper we will refer with the term reads to the short reads returned by the Illumina Sequencing Technology

## References

[1] Buermans HPJ, den Dunnen JT. Next Generation Sequencing Technology: Advances and Applications. Biochim Biophys Acta (BBA) - Mol Basis Dis. 2014;1842(10):1932–1941. From genome to function. 10.1016/j.bbadis.2014.06.015. 10.1016/j.bbadis.2014.06.015 24995601

[2] Di Rocco L, Ferraro Petrillo U. A Distributed Alignment-free Pipeline for Human SNPs Genotyping. In: Proceedings of the 14th ACM International Conference on Bioinformatics, Computational Biology, and Health Informatics; 2023. pp. 1–8. Association for Computing Machinery, New York, United States.

[3] Shajii A, Yorukoglu D, William YuY, Berger B. Fast Genotyping of known SNPs through Approximate k-mer Matching. Bioinformatics. 2016;32(17):i538–44. 27587672 10.1093/bioinformatics/btw460 PMC5013917

[4] Sun C, Medvedev P. Toward Fast and Accurate SNP Genotyping from Whole Genome Sequencing Data for Bedside Diagnostics. Bioinformatics. 2019;35(3):415–20. 30032192 10.1093/bioinformatics/bty641

[5] Denti L, Previtali M, Bernardini G, Schönhuth A, Bonizzoni P. MALVA: Genotyping by Mapping-free ALlele Detection of Known VAriants. iScience. 2019;18:20–27. RECOMB-Seq 2019. 10.1016/j.isci.2019.07.011. 10.1016/j.isci.2019.07.011 PMC6664100 31352182

[6] Khorsand P, Hormozdiari F. Nebula: Ultra-efficient Mapping-free Structural Variant Genotyper. Nucleic Acids Res. 2021;49(8):e47–e47. 33503255 10.1093/nar/gkab025 PMC8096284

[7] Ebler J, Ebert P, Clarke WE, Rausch T, Audano PA, Houwaart T, et al. Pangenome-based Genome Inference Allows Efficient and Accurate Genotyping across a Wide Spectrum of Variant Classes. Nat Genet. 2022;54(4):518–25. 35410384 10.1038/s41588-022-01043-w PMC9005351

[8] Iqbal Z, Caccamo M, Turner I, Flicek P, McVean G. De Novo Assembly and Genotyping of Variants using Colored De Bruijn Graphs. Nat Genet. 2012;44(2):226–32. 22231483 10.1038/ng.1028 PMC3272472

[9] Leggett RM, Ramirez-Gonzalez RH, Verweij W, Kawashima CG, Iqbal Z, Jones JD, et al. Identifying and Classifying Trait Linked Polymorphisms in Non-Reference Species by Walking Coloured De Bruijn Graphs. PLoS ONE. 2013;8(3):e60058. 23536903 10.1371/journal.pone.0060058 PMC3607606

[10] Muggli MD, Bowe A, Noyes NR, Morley PS, Belk KE, Raymond R, et al. Succinct Colored De Bruijn Graphs. Bioinformatics. 2017;33(20):3181–7. 28200001 10.1093/bioinformatics/btx067 PMC5872255

[11] Bifrost: Highly Parallel Construction and Indexing of Colored and Compacted De Bruijn Graphs, author=Holley, Guillaume and Melsted, Páll. Genome Biol. 2020;21(1):1–20. 10.1186/s13059-020-02135-8 PMC7499882 32943081

[12] Durbin R. Efficient Haplotype Matching and Storage using the Positional Burrows-Wheeler Transform (PBWT). Bioinformatics. 2014;30(9):1266–72. 24413527 10.1093/bioinformatics/btu014 PMC3998136

[13] Uricaru R, Rizk G, Lacroix V, Quillery E, Plantard O, Chikhi R, et al. Reference-free Detection of Isolated SNPs. Nucleic Acids Res. 2015;43(2):e11–e11. 25404127 10.1093/nar/gku1187 PMC4333369

[14] Drezen E, Rizk G, Chikhi R, Deltel C, Lemaitre C, Peterlongo P, et al. GATB: Genome Assembly & Analysis Tool Box. Bioinformatics. 2014;30(20):2959–61. 24990603 10.1093/bioinformatics/btu406 PMC4184257

[15] Peterlongo P, Riou C, Drezen E, Lemaitre C. DiscoSnp++: De Novo Detection of Small Variants from Raw Unassembled Read Set(s). BioRxiv. 2017:209965.

[16] Gauthier J, Mouden C, Suchan T, Alvarez N, Arrigo N, Riou C, et al. DiscoSnp-RAD: De Novo Detection of Small Variants for RAD-Seq Population Genomics. PeerJ. 2020;8:e9291. 32566401 10.7717/peerj.9291 PMC7293188

[17] Dean J, Ghemawat S. MapReduce: Simplified Data Processing on Large Clusters. Commun ACM. 2008;51:107–13.

[18] Apache Software Foundation. Apache Spark, 2016. http://spark.apache.org. Accessed 1 May 2023.

[19] Xin RS, Gonzalez JE, Franklin MJ, Stoica I. Graphx: a Resilient Distributed Graph System on Spark. In: First international workshop on graph data management experiences and systems, 2013. pp. 1–6. Association for Computing Machinery, New York, United States.

[20] Di Rocco L, Ferraro Petrillo U, Rombo SE. DIAMIN: a software library for the distributed analysis of large-scale molecular interaction networks. BMC Bioinformatics. 2022;23(1):474. 36368948 10.1186/s12859-022-05026-w PMC9652854

[21] Malewicz G, Austern MH, Bik AJ, Dehnert JC, Horn I, Leiser N, et al. Pregel: a System for Large-scale Graph Processing. In: Proceedings of the 2010 ACM SIGMOD International Conference on Management of data, 2010. pp. 135–46. Association for Computing Machinery, New York, United States.

[22] Chikhi R, Limasset A, Medvedev P. Compacting De Bruijn Graphs from Sequencing Data Quickly and in Low Memory. Bioinformatics. 2016;32(12):i201–8. 27307618 10.1093/bioinformatics/btw279 PMC4908363

[23] Ferraro Petrillo U, Roscigno G, Cattaneo G, Giancarlo R. FASTdoop: a Versatile and Efficient Library for the Input of FASTA and FASTQ Files for MapReduce Hadoop Bioinformatics Applications. Bioinformatics. 2017;33(10):1575–7. 28093410 10.1093/bioinformatics/btx010

[24] Amorosi L, Di Rocco L, Ferraro Petrillo U. Scheduling k-mers Counting in a Distributed Environment. In: Optimization in Artificial Intelligence and Data Sciences: ODS, First Hybrid Conference, Rome, Italy, September 14-17, 2021. Springer; 2022. pp. 73–83.

[25] Besta M, Kanakagiri R, Mustafa H, Karasikov M, Rätsch G, Hoefler T, Solomonik E. Similarity Communication-efficient Jaccard, for High-Performance Distributed Genome Comparisons. In: 2020 IEEE International Parallel and Distributed Processing Symposium (IPDPS). IEEE; 2020. pp. 1122–32.

[26] Rasheed Z, Map-Reduce Rangwala H. A, Framework for Clustering Metagenomes. In: 2013 IEEE International Symposium on Parallel & Distributed Processing, Workshops and Phd Forum. IEEE; 2013. pp. 549–58.

[27] Zook JM, Chapman B, Wang J, Mittelman D, Hofmann O, Hide W, Salit M. Integrating Human Sequence Data Sets Provides a Resource of Benchmark SNP and Indel Genotype Calls. Nat Biotechnol. 2014;32(3):246–51. 10.1038/nbt.2835 24531798

[28] Bompiani E, Ferraro Petrillo U, Lasinio GJ, Palini F. High-Performance Computing with TeraStat. In: 2020 IEEE Intl Conf on Dependable, Autonomic and Secure Computing, Intl Conf on Pervasive Intelligence and Computing, Intl Conf on Cloud and Big Data Computing, Intl Conf on Cyber Science and Technology Congress (DASC/PiCom/CBDCom/CyberSciTech). Los Alamitos: IEEE Computer Society; 2020. pp. 499–506. 10.1109/DASC-PICom-CBDCom-CyberSciTech49142.2020.00092.

[29] Deorowicz S, Kokot M, Grabowski S, Debudaj-Grabysz A. KMC 2: Fast and Resource-frugal k-mer Counting. Bioinformatics. 2015;31(10):1569–76. 25609798 10.1093/bioinformatics/btv022

